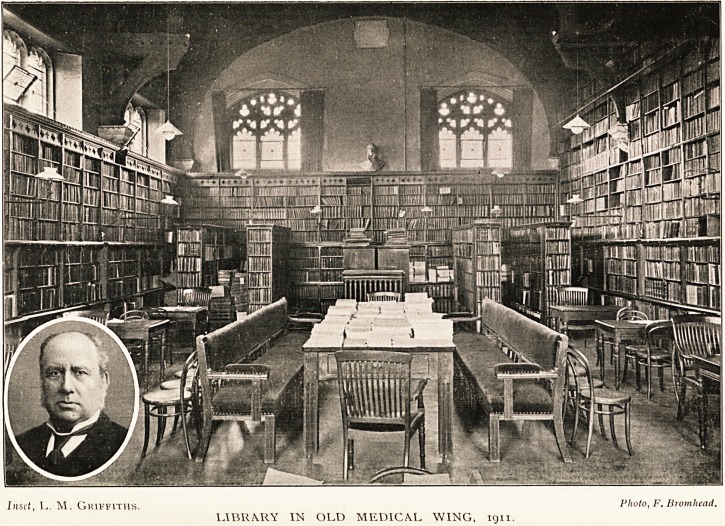# The Bristol Medical Library

**Published:** 1911-12

**Authors:** L. M. Griffiths

**Affiliations:** Honorary Librarian to the Library of the Bristol Medico-Chirurgical Society (1890-93) and to the Bristol Medical Library (1893-1902)


### THE BRISTOL MEDICAL LIBRARY.

BY

L. M. Griffiths, M.R.C.S. Eng., L.R.C.P. Ed.,

*Honorary Librarian to the Library of the Bristol Medico-Chirurgical Society* (1890–93) *and to the Bristol Medical Library* (1893–1902).

It is not till the beginning of the nineteenth century that there is any evidence that Bristol doctors felt the necessity of co-operation for study of books that would be useful to them in their work, and which it would be beyond their power to possess individually.

But in the first decade of that period there were in Bristol two flourishing Medical Reading Societies, the earlier of which has long ceased to be, while the other,[1] founded in 1807, has had a continuous existence to the present day, and is now, at least, as vigorous as ever, and is exceedingly popular.

Such Societies, however, admirable and useful as they are, have unavoidable limitations, as they are compulsorily restricted to only a few members. Therefore we find that in 1831[2] the need for more extended opportunities was met by the formation of the Bristol Medical Library, and in 1832 its home was in Orchard Street, a most convenient situation, as a large number of medical men were living in the immediate neighbourhood.

The building was that which had been used for worship by a body of French Protestants who, after the revocation of the Edict of Nantes by Louis XIV in 1685, had settled in Bristol

[1] For the history of this Society, see *Bristol M.-Chir. J.*, 1907, xxv. 222–36.

[2] See *Felix Farley's Bristol Journal*, August 13th, 1831.

in large numbers, and who at their first coming had been permitted by the Corporation to use the Mayor's Chapel [1] for their weekly services. But when the city authorities decided to use the chapel for themselves they granted, in 1729, a plot of ground to the Protestants for the erection of a building for their own use, and it was thus occupied till 1825, when their numbers had so dwindled that a building was no longer required.

In 1832 the Corporation granted the use of the premises to Dr. Kentish, Dr. Davies, and Mr. Mortimer [2] at a yearly rent of two pounds for a medical library. Part of the negotiations with the Corporation were carried on by Dr. Stanton. [3] The building still stands, and is now used for service by the Plymouth Brethren, who have had it since 1856.

Kentish, the founder of the Library and a generous contributor to it, was the first President, and held office till his death on December 8th, 1832. His successor was Dr. Carrick. [4]

At the fifth annual meeting on June 27th, 1836, [5] the Library contained 1,200 volumes, and it was reported that papers had been read at evening meetings, so that it is evident that the

[1] The Corporation at that time attended the Cathedral at their ordinary services.

[2] Edward Kentish, living at 27 Park Street, was physician to St. Peter's Hospital and the Dispensary. His name is familiar to the present generation in connection with the Kentish Baths, opposite the east end of the Cathedral. Kentish was President of the Anchor (Colston) Society in 1828.

David Davies, who lived at 17 Park Street, was surgeon to St. Peter's Hospital, and lectured on medicine at the School in College Green.

William Mortimer, in practice at 33 Richmond Terrace, was an original member of the 1807 Reading Society, in which he continued until 1840. He was President of the Dolphin (Colston) Society in 1832.

[3] John Stanton, M.D., lived at 1 Richmond Hill. He was physician at the General Hospital from 1854 to 1856.

[4] Andrew Carrick, M.D., living then in Clifton Hill, was at the time Senior Physician at the Infirmary. He resigned in 1834, having held office for twenty-four years. At the election in 1810 he, the representative of the Tories, was successful by 448 votes against 216 recorded for the Whig candidate, Dr. J. E. Stock, the biographer of Beddoes, and who became President of the Anchor (Colston) Society in 1816. Dr. J. C. Prichard was a third candidate, receiving 81 votes. In 1833 Carrick was President of the Bristol meeting of the Provincial Medical and Surgical Association (founded in 1832), now the British Medical Association. Carrick, at the time of his death in 1837, was President-elect of the Dolphin (Colston) Society.

[5] See *Felix Farley's Bristol Journal*, July 2nd, 1836.

*Inset*, L. M. GRIFFITHS. *Photo, F. Bromhead.*

LIBRARY IN OLD MEDICAL WING, 1911.

members were not confining themselves to Library work, and this explains the title of Bristol Medical Library Society, by which the institution was then known.

At the annual meeting on June 26th, 1837, with David Davies in the chair, it was necessary to appoint a new president in the place of Dr. Carrick, whose death had occurred on the 14th. Dr. Symonds,[1] in a most eloquent speech, after referring appropriately to the merits of Carrick, proposed the election of Dr. James Cowles Prichard[2] in exquisitely chosen phrases, which deserve the attention of every reader of the present day.[3] This was seconded by Mortimer. The Society re-elected Dr. Paris Dick[4] as secretary, Mr. Surrage[5] as treasurer, and Mr. Green[6] as librarian.

The institution known as the Bristol Library Society, formed in 1772 on subscription lines, had, through the negligence of the city authorities, usurped the building which in 1740 had replaced the house in King Street given in 1613 by Richard Redwood to the city for the purpose of a library for public use.

This anomalous state of things continued till 1854, when, as a result of the protest of some citizens, the King Street building was re-established for its original object, and the Bristol Library Society, having changed its title to that of the Bristol Library, moved in 1855 to the west wing of the Bishop's College, which stood on ground now occupied by the Art Gallery.

[1] John Addington Symonds, M.D., then living at 15 Park Street, was one of the physicians at the General Hospital. He had been elected at the time of its foundation in 1832, and retained office till 1844. He was also in 1837 one of the Dispensary physicians.

[2] He was living at the Red Lodge in Park Row, and was Senior Physician at the Infirmary, having been elected in 1816. He held office till 1843.

[3] The speech is reported at length in *Felix Farley's Bristol Journal*, and in *The Bristol Mirror* of July 1st, 1837.

[4] P. T. Dick, M.D., Beaufort Buildings, Clifton.

[5] Thomas Lyddon Surrage, Surgeon, 2 York Buildings, Clifton.

[6] Thomas Green, Surgeon, 9 Queen Square. He was surgeon at the Infirmary from 1844 to 1864. In 1843, upon the death of Richard Smith, he had lost the election with 283 votes against Henry Clark's 521. He was a Town Councillor from 1842 to 1853, an Alderman from 1853 to 1877, and died in 1878. In 1853 he was President of the Dolphin (Colston) Society.

In 1855 negotiations were begun for the transfer of the Medical Library to the custody of the Bristol Library, and in 1856 the transfer took place on conditions to which the Bristol Library consented in the following terms :—

(1) That the existing members of the Bristol Medical Library, being about forty in number, or such of them as shall assent thereto, shall be admitted as members of this Library on the footing of life members, paying each an annual subscription of one guinea and a half.

(2) That of this annual subscription the sum of fifteen shillings shall be applied to the purchase ot medical works.

(3) That for the purpose of securing the best appropriation of these sums not less than three members of the medical professon being members of this Society shall be elected annually to be members of the general committee.

On reporting these conditions to a general meeting the Committee said that they believed "that the introduction of so considerable and influential a body of subscribers as will be added to the Bristol Library Society will largely promote the interest and prosperity of this Society, and considering the terms proposed to be liberal and advantageous, strongly recommend to this special general meeting to accept and confirm this report, and to give power to the Committee to effect the union of the two Societies on the terms proposed."

The report was adopted, and in 1856 the books were transferred. In March of that year the doctors passed over £15, the balance of their Library Fund, to the Bristol Library.

By March, 1857, thirty-eight medical works had been added, and in April it was decided to insure the whole medical collection against fire for £600. In November the medical duplicates were sold to the General Hospital.

In March, 1859, the Library was indebted £17 15s. 2d. to the fund allotted for medical works, and it was determined to take steps to diminish general expenses.

The amount of indebtedness to the medical fund stood at £19 14s. 1d. in 1860, when £29 15s. 1d. had been spent in medical works.

In 1861 the insurance on the medical portion was reduced to £300. The doctors voted £15 15s. to the general library towards an increased subscription to Mudie's of £52 10s., accepting only £16 worth of books for the year instead of the £31 15s. to which they were entitled by the original terms. An appeal to Bishop Monk, the trustee of the property, for a reduction of the rent from £100 to £80, as in the first two years, failed. The sale of the building was contemplated.

The doctors upon being appealed to in 1862 for a renewal of their contribution to the subscription to Mudie's granted it. The Bishop's College had ceased to exist in 1861, and the property then passed to the ownership of the Bristol Rifles Head-quarters Company, which, proposing certain alterations, offered to reduce the rent by £20. Owing to the changes in the tenancy, the question of raising a fund for the purchase of premises was brought forward.

In 1863 this assumed a more definite form, as a resolution was received from the Bristol Philosophic and Literary Institution, which, founded in 1817, was carrying on its excellent work in Park Street in the building now known as the Freemasons' Hall. This resolution, which suggested union with the Library in a new building, was welcomed, and a sub-committee appointed to consider it as a practical measure. The doctors voted in March of this year a further contribution of seven guineas and a half for the Mudie subscription, but with an intimation that this was to be the last of the kind.

The question of amalgamation of the two institutions was delayed in 1864, and the Rifles gave the Library notice to quit in March, 1865, but a prolonged stay was arranged under different conditions. The Library was still bent upon acquiring premises of its own, and now proposed a plan in which one room was to be devoted to the Medical Library.

Negotiations in 1865 with the Victoria Rooms Company came to nothing, and the proposal for amalgamation with the Philosophical Institution took further shape and was finally arranged.

In 1866 the Library decided to make a general appeal to doctors to become members of the Library.

The insurance of the medical collection was raised to the original amount of £600 in 1867, in October of which year the medical members ordered the sale of their microscope.

Notwithstanding many difficulties, the project of a united building for the two institutions was carried on, and eventually in 1871 the Museum and Library in Queen's Road was ready for their joint occupation, and with this the Medical Library found a new home. Its condition there is familiar to many of us, and whilst we are ready to admit that it was rich in bygone literature, it was in no way adequate for contemporary needs.

The full records of the Museum and Library are not available, but from some of the annual reports it would seem that the medical portion of the Library had no definite recognition, although in that of 1877 there is a project of placing in an appendix to the general catalogue a list of the medical works, and in that of 1878 it is stated that a separate catalogue of the medical books was in preparation.

In the statement of accounts there is no separation of the amounts spent in the purchase of medical books as in the earlier days, when the amounts were as follows :—

| | | | | | | | |
|---|---|---|---|---|---|---|---|
| 1857 .. .. | £28 | 2 | 0 | 1865 .. .. | £20 | 18 | 0 |
| 1858 .. .. | 22 | 9 | 10 | 1866 .. .. | 17 | 4 | 3 |
| 1859 .. .. | 18 | 16 | 0 | 1867 .. .. | 20 | 10 | 8 |
| 1860 .. .. | 29 | 15 | 1 | 1868 .. .. | 15 | 16 | 0 |
| 1861 .. .. | 24 | 14 | 0 | 1869 .. .. | 14 | 16 | 0 |
| 1862 .. .. | 19 | 11 | 3 | 1870 .. .. | 18 | 9 | 6 |
| 1863 .. .. | 19 | 1 | 4 | 1871 .. .. | 17 | 2 | 3 |
| 1864 .. .. | 20 | 13 | 0 | | | | |

The Museum and Library did not receive the public support it expected, and after a languishing career it passed in 1893

(through the generosity of the shareholders and the liberality of Sir Charles Wathen, who undertook the payment of all outstanding liabilities) to the ownership of the city.

Before this, the establishment[1] of the *Bristol Medico-Chirurgical Journal,* under the auspices of the Bristol Medico-Chirurgical Society,[2] which had been founded in 1874 mainly through the efforts of Dr. Shingleton Smith, had opened out new possibilities. From its beginning in 1882 it had received a large number of exchange-journals and many books for review. At first the periodicals were materially dissected for the convenience of those who wrote the medical periscopes, and the books became the property of those who reviewed them. In course of time a general feeling arose that much valuable matter was being lost to the majority of the members of the Society, and the desirability of forming a library was brought before the Society on February 8th, 1888, when the question

[1] For notes on this see *Bristol M.-Chir. J.*, 1891, ix. 243, and 1900, xviii. 101.

[2] Other societies bearing somewhat similar titles had preceded it. In 1812 serious unascertainable differences arose in the 1807 Reading Society (see *Bristol M.-Chir. J.*, 1907, xxv. 227). Daniel (who was surgeon at the Infirmary 1810–36, and Master of St. Stephen's Ringers in 1817) and Edgell (who in 1812 was living at 2 College Street, afterwards removing to 17 College Square, now Lower College Green, where his name is still to be seen on the garden wall of the present Deanery) resigned in March, Lax and Nathaniel Smith in April, and Crang in May. These, with eleven who did not belong to the Reading Society, decided to form "The Medical and Chirurgical Society," to consist of twelve members, having the same objects as the 1807 Society—in fact, merely another Reading Society, with the added attraction of a supper at each meeting.

On the initiative of the Infirmary doctors, who constituted the Committee, "The Medical and Chirurgical Association" was formed in 1819 to deal with ethical matters and the question of fees. The members were to meet twice a year and dine. Richard Smith (surgeon to the Infirmary from 1796 to 1843, when he died, and to whom the profession of to-day is so largely indebted for his scientific zeal and literary industry) was secretary, but resigned in the following year. He was President of the Dolphin (Colston) Society in 1822, and Master of St. Stephen's Ringers in 1826.

The meeting on June 18th, 1840, when it was decided to adopt the proposal of June, 1839, to form a Bristol branch of the Provincial Medical and Surgical Association, now the British Medical Association, was held in the Orchard Street Medical Library. (See *Felix Farley's Bristol Journal* and *Bristol Mirror,* June 20th, 1840, and *Bristol Gazette* and *Bristol Standard,* June 25th, 1840.) Cowles Prichard was the first President and Richard Smith the next. G. H. Hetling was secretary from 1839 to 1847. The proceedings at a dinner held after the meeting lasted from half-past five till midnight. The Bath branch had been formed in 1836. The two were united in 1841. (See *Brit. M. J.*, 1882, i. 956.)

was relerred to the committee to report. They came to the conclusion (1) that a library is desirable; (2) that it should be in conjunction with the Medical School; and (3) that inquiries be made as to what temporary plans can be formed pending the erection of new Medical School buildings.

No definite result followed this, but there was a widespread feeling amongst the members generally that a beginning should be made irrespective of the Medical School; and on May 14th, 1890, the matter came again before the Society which passed *nem. con.* an instruction to the committee "to present at the October meeting a formulated scheme for a Medical Library to be founded under the auspices of the Society."

The committee, meeting on June 7th, received a communication signed by fifty-four members of the Society requesting that arrangements should be made for enabling the members to consult the medical periodicals received through the *Journal*, and for the formation of a Medical Reading Room. At the same time application came from other local scientific societies asking for co-operation in founding a club which should have for one of its objects the gathering together of all these societies under one roof. From these communications followed the establishment, at 28 Berkeley Square, of the Literary and Philosophic Club, where accommodation was offered to the Society, and this proposal met with the unanimous approval of the committee on September 24th, 1890, and they recommended to the Society the following scheme, which was adopted at the general meeting in October:—

(1) The Society shall rent a room or rooms for the purpose of a Medical Reading Room and Reference Library to be known as "The Library of the Bristol Medico-Chirurgical Society."

(2) The Library shall be open not earlier than 10.0 a.m., and not later than 11.0 p.m., and shall be free to all members of the Society.

(3) It shall be managed by the committee of the Society, who shall annually appoint an Honorary Librarian.

(4) All exchange-journals and books[1] received through the Society's *Journal* shall be placed in the Library.

(5) All books and periodicals therein shall be in the custody of the Honorary Librarian, who shall be responsible for the due care of the same.

(6) A book shall be kept in the room to receive suggestions from members for increasing the usefulness and comfort of the Library. All such suggestions shall be considered by the committee at their next meeting, and answers thereto entered in the book.

A sub-committee was appointed to carry out details, and on January 5th, 1891, amid comfortable and well-appointed surroundings, the President of the Society formally opened "The Library of the Bristol Medico-Chirurgical Society." By the aid of generous gifts of money and books (including a large donation from the British Medical Association), a start was made with over a thousand volumes and seventy-nine current journals. It had been decided that *active* canvassing for the Library cease for the time, and that it be transferred under proper guarantee to the Library in the new Medical School Buildings. The Librarian was empowered to spend in the purchase of books, in addition to the amounts collected for Library purposes, a sum not exceeding £100 from the accumulated profits of the *Journal.*

In 1891 the conduct of the *Journal*, which hitherto had been in the hands of the Editor and Assistant-Editor, was passed over to a Committee which consisted of these and four others to work in co-operation. The value of the *Journal* as a handmaid to the Library has been constant, and can scarcely be overrated.

At Berkeley Square the Library remained till July 1st, 1893, when the books were moved into a magnificent apartment

[1] The achievement of this most desirable object was rendered easy by the exemplary self-denial of the reviewers of books, who consented that the volumes should no longer be their property, but should find a place in the Library for consultation.

specially constructed for library purposes in the Medical School, The new building had been opened on November 16th, 1892, and in the meantime an arrangement for a joint library had been made with University College,[1] which did much to make the scheme a success, and contributed greatly to the convenience of the members. The management was vested in a committee consisting of three representatives from the Medical Faculty of the College and three from the Society. The first Chairman was Greig Smith, who took the warmest interest in the development of the Library.

The Medical School had no library, but books in large numbers soon came in, and both sections of the Library grew rapidly. In 1891 the Society had 1,879 books and 79 current periodicals. In 1901 the numbers were 9,371 and 189 respectively, and now at the end of another period of ten years the figures are 11,619 and 190. While owing to the advantages of the conjoint scheme members of the Society can consult 21,929 volumes and 249 periodicals. The progress of the Library is fully chronicled in detail in the annual reports presented to the Society, and printed in the *Bristol Medico-Chirurgical Journal.*

Through the influence and energy of Greig Smith nearly all the books belonging to the Infirmary and the Hospital were added in 1894, and, later on, the medical books from the Museum and Library, which, of course, included many from the old Orchard Street collection, were passed on by the city authorities to become once more taken care of in a reconstituted "Bristol Medical Library."

[1] This, with some of its after-modifications, is printed in the Society's booklet recently issued.

### BIBLIOGRAPHY OF THE BRISTOL MEDICAL LIBRARY.

*Felix Farley's Bristol Journal*, August 13th, 1831; July 2nd, 1836; July 1st, 1837.

*New Monthly Magazine*, September, 1831.

*Bristol Mirror*, July 1st, 1837.

*Bristol M.-Chir. J.*, 1890, viii. 287; 1891, ix. 66, 215, 246, 313; 1892, x. 284, 332; 1893, xi. 282; 1894, xii. 330; 1895, xiii. 316; 1896, xiv. 373; 1897, xv. 367; 1898, xvi. 369; 1899, xvii. 368; 1900, xviii. 102; 1901, xix. 284, 376; 1902, xx. 376; and later references.

## Figures and Tables

**Figure f1:**